# Scalable biclustering — the future of big data exploration?

**DOI:** 10.1093/gigascience/giz078

**Published:** 2019-06-28

**Authors:** Patryk Orzechowski, Krzysztof Boryczko, Jason H Moore

**Affiliations:** 1 Institute for Biomedical Informatics, University of Pennsylvania, 3700 Hamilton Walk, Philadelphia, PA 19104, USA; 2 Department of Automatics and Robotics, AGH University of Science and Technology, al. A. Mickiewicza 30, Kraków 30-059, Poland; 3 Department of Computer Science, AGH University of Science and Technology, al. A. Mickiewicza 30, Kraków 30-059, Poland

**Keywords:** biclustering, co-clustering, data mining, big data, parallel algorithms, disease subtype identification, biomarker detection, gene-drug interaction, precision medicine

## Abstract

Biclustering is a technique of discovering local similarities within data. For many years the complexity of the methods and parallelization issues limited its application to big data problems. With the development of novel scalable methods, biclustering has finally started to close this gap. In this paper we discuss the caveats of biclustering and present its current challenges and guidelines for practitioners. We also try to explain why biclustering may soon become one of the standards for big data analytics.

## Background

The volume of data is rapidly growing, especially in the biomedical domain. In recent years multiple scientific projects have produced large-scale data. In the 100,000 Genomes Project [https://www.genomicsengland.co.uk/about-genomics-england/the-100000-genomes-project], 100,000 whole genomes from National Health Service patients were sequenced in the United Kingdom with focus on rare diseases, infectious diseases, and cancer. A similar effort is being taken all across the world [https://www.clinicalomics.com/topics/biomarkers-topic/biobanking/10-countries-in-100k-genome-club]. One million individuals are expected to participate in a recently launched "All of Us" initiative in the United States [https://allofus.nih.gov]. Their genetic and health data will be gathered to foster collaborative research on delivering precision medicine addressing different lifestyles and a wide range of health conditions.

In the era of big data, information retrieval becomes key. There is an emerging need for developing tools that could face the challenge of large amounts of data. The methods are expected to be not only precise but also tolerant to noise, scalable, and fast. The results are expected to be interpretable in order to provide a better understanding of underlying structures in the data. Moreover, the tools are required to capture local similarities in the data, which reflect high heterogeneity.

One of the areas of research in which great progress has been made in recent years to address the aforementioned big data challenges is biclustering [1–4]. This analytical technique of data mining, which is also known as subspace clustering, co-clustering, block clustering, or 2-mode clustering, has already become an essential tool for gene expression analysis because it is capable of capturing similar gene expression profiles under different subsets of experimental conditions [5]. It is not without reason that biclustering has found hundreds of applications in bioinformatics, and, as a result, there has been a call for increased use of this approach [6]. The era of biclustering big data has begun.

## What is Biclustering?

There are multiple formulations of the biclustering problem, so as to meet multiple challenges. Generally, biclustering is the task of identifying a single or many biclusters, where each of the biclusters is a subset of rows with similar behavior across a subset of columns (or vice versa). For a dataset *A* = [*a_ij_*]_*m* × *n*_ with rows denoted as *X* = (*x*_1_, *x*_2_, …, *x_m_*) and columns as *Y* = (*y*_1_, *y*_2_, …, *y_n_*), biclustering is an identification of a single or a series of *p* biclusters *B_k_* = (*I_k_*, *J_k_*), where *k* = 1, 2, …, *p; I_k_* ⊆ *X* are the rows of the *k*th bicluster; and *J_k_* ⊆*Y* are the columns of the *k*th bicluster, where each of the biclusters meets some homogeneity criteria [7]. Biclustering could also be viewed from different angles as detection of sub-matrices, cliques in a bipartite graph, or communities.

**Figure 1.**
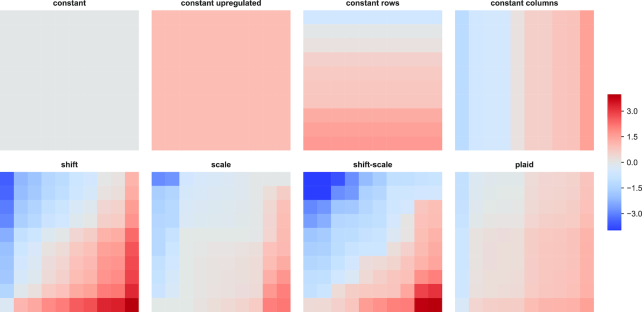
Different patterns in biclustering. The original patterns were sorted first by rows and second by columns for visualization purposes. Biclusters with constant or upregulated pattern have all values exact. Constant rows/columns patterns are characterized by the same value across all columns/rows of the bicluster. The values between rows/columns may differ. In a bicluster with shift pattern the contribution of a given row is added to the contribution of a given column, whilst in the case of scale pattern the contribution of a row is multiplied. In a shift-scale pattern each row contributes twice: by a factor that is multiplied by a column contribution and by an additive further shifting the values. In plaid patterns, the data are modeled as a sum of multiple layers. Note that all the patterns could be considered order-preserving.

Although biclustering is generally considered an unsupervised machine learning technique, multiple semi-supervised or supervised approaches have been proposed that are based on related concepts. Biclustering is closely related to fuzzy clustering and frequent itemset mining, as well as learning classifiers systems. Depending on the field of application, the data for the algorithms could be numerical (binary, discrete, or continuous), categorical, or ordinal. The methods may attempt to detect a single bicluster, exclusive biclusters (their rows, columns, or both may belong to ≤1 bicluster), disjoint biclusters (i.e., non-overlapping, e.g., checkerboard), inclusive biclusters (the only overlap between biclusters could be inclusion), or arbitrary positioned biclusters. The patterns to be detected can also vary, starting from classical biclustering problems (constant values, upregulated values, constant values in rows, constant values in columns, shift patterns, scale patterns, shift-scale patterns, plaid patterns, order-preserving−coherent evolutions) [8]. Some of the most popular data patterns for biclustering generated using BiBench [ http://tda.gatech.edu/software/bibench-v0.2] are presented in Figure   1.

### What is the application of biclustering?

Biclustering has been successfully applied to hundreds of problems in the biological and biomedical domain and has supported detection of functional annotations (e.g., gene regulatory pathways), as well as biological interactions (e.g., transcriptional networks), the discovery of drugs and biomarkers, identification of subtypes of diseases, and analysis of responses to treatments [6]. A biclustering method has also helped to identify novel human microRNA regulatory modules [2]. Biclustering techniques have been also been successfully used in graph analysis, text mining, recommendation systems, marketing, economy (e.g., market segmentation), analysis of sports data, and multiple other domains [1]. Among multiple domains, gene expression data are commonly considered a real data playground for measuring the performance of biclustering methods.

### Common myths about biclustering

Let us demystify some common views on biclustering.

#### Is biclustering a local equivalent of clustering?

This is correct. While clustering looks for global similarities within data and takes into consideration all data dimensions, biclustering captures patterns hidden locally and uses only some of the data dimensions.

#### Is biclustering the same as 2-way clustering?

Not necessarily. Although some of the first biclustering methods used to cluster first by rows, then by columns (or the other way around), the field has progressed far since its emergence. Usually, biclustering techniques use information either from both rows and columns at the same time, or alternately from rows and columns to progress.

#### Biclustering = feature selection + clustering?

Although it seems likely, this is not true. Usually different features contribute to different biclusters. There are some common aspects though, as for each bicluster certain features are selected. The closest answer is that biclustering borrows from both techniques but is certainly not a combination of both.

#### Is biclustering a dimensionality reduction technique?

The answer is no, but biclustering can be used as a technique that reduces dimensionality because it finds patterns with subsets of rows and columns with very similar characteristics.

#### Biclustering is neither generative nor predictive.

True. Biclustering algorithms are usually expected to retrieve existing (but hidden) information and thus provide insight into data. The methods are not intended to generate the data, nor to make predictions. Biclustering methods are intended to locate specific patterns, which they were designed for.

#### Biclustering is much more complex than deep learning.

The majority of problems in biclustering are considered NP-complete [7]. To better visualize the complexity of usual biclustering problem, let us consider a task of detecting an object on an image. The biclustering task would be formulated as finding the same object, but in the image with randomly shuffled rows and randomly shuffled columns. The assumption that neighboring rows or columns belong to the same object, or that 2 related objects are next to each other (e.g., words creating context), greatly simplifies the problem and makes deep learning methods much more efficient for image analysis, or natural language processing tasks.

## Biclustering and Big Data

The biclustering field has largely evolved since its first application to gene expression in 2000. Modern methods take advantage of parallel computation or the map-reduce paradigm. The popular environments for launching large-scale biclustering analyses are becoming Hadoop [https://hadoop.apache.org], Apache Spark [https://spark.apache.org], and massively parallel systems with multiple GPUs [5].

Recently, a very accurate and scalable method for biclustering big data called EBIC was proposed for multi-GPU environments [3,4]. This open source method [https://github.com/EpistasisLab/ebic] manages to detect multiple patterns in the data and scales very well for large datasets. Its latest release allows missing values to be omitted, which makes the method applicable to RNA sequencing (RNA-seq) and single-cell RNA-seq (scRNA-seq) data.

### Scalable biclustering

With continuously increasing sizes of the data many traditional biclustering approaches struggle to analyze the data within a reasonable time frame. Large data volume and high problem complexity, as well as poor memory management of some of the implementations, make a lot of approaches not feasible for handling big data problems. Thus, one of the main focuses of future algorithm design is their scalability. By "scalability" we understand the ability of the method to handle increasing sizes of the data with additional resources (e.g., CPUs, GPUs, or TPUs) in a reasonable time.

Considering the algorithm design, it is crucial to understand where and how parallelization in the method may be exploited to provide speedup, so desirable for analyzing large datasets [5]. Alternatively, the development of a new method could start with understanding hardware limitations. From our experience, the second option is more extendable (e.g., EBIC was designed from scratch in compliance with GPU memory limitations and programming constraints).

### Challenges of biclustering

Apart from the ability to analyze big data, we would like to summarize some of the major challenges that biclustering currently faces.

#### How many biclusters?

The larger gets the volume of the data, the greater is the number of potential solutions. Usually biclustering methods yield either the requested number or (by default) up to 100 biclusters. Some methods, however, may return even millions of patterns. From the perspective of the end user, performing analysis or validation of that many candidates becomes extremely challenging. Because each scenario varies, we believe this should be up to the user to determine a reasonable number of biclusters that would be suitable for their purpose. One of the unexplored caveats of biclustering is that the number of expected biclusters actually influences which patterns should be indicated as biclusters, which is especially visible in partially overlapping scenarios. If the method does not account for the user indication, we recommend reporting biclusters with the highest relevance before the others (e.g., by maintaining a ranked list of the best solutions), so that the most important solutions are not missed if the user requests only a couple of biclusters, as well as filtering out highly overlapping biclusters.

#### What should be the sizes of biclusters?

It is an open-ended question what sizes of the patterns (local or global? narrow or wide?) are more relevant, and the answer probably depends on the specific domain of application. For some scenarios (e.g. detection of cohorts), the larger sizes of biclusters are preferable; for other (e.g. gene enrichment analysis) - not necessarily. Big data is definitely not helping here—this task may resemble looking for a needle in a haystack if the objective is to find a small correlated pattern.

#### How should the performance be measured?

The most established measures in the field, called recovery and relevance, are based on the Jaccard index and were shown to be inadequate for objective assessment of the performance of biclustering methods. Horta and Campello reviewed different measures for biclustering and presented their desirable properties [9]. Biclustering measures should increasingly penalize noisy entries or elements not found in both biclusterings. Not covering all solutions, reporting elements not belonging to a specific bicluster, as well as covering the same elements multiple times should also be penalized. Repetitively reporting a very similar bicluster is also not desirable. Finally, the measure should be symmetric and return score equal to 1 for a perfect fit. Although the authors reported 2 measures that have the desired properties, only CE [10] does not penalize heterogeneous patterns, which are very common in genomics. Thus, we believe that CE should be considered as the most objective measure of performance of biclustering methods for all synthetic scenarios.

### Interpretability is the key

In the biomedical domain, interpretation is performed using expert knowledge. Gene set enrichment analysis and pathway analysis are common techniques of validation. One of the major advantages of biclustering over many other methods (e.g., feature selection) is interpretability of the results. Biclusters are much easier to interpret because they extract very specific patterns, for detection of which the given method was designed [8]. Interpretability greatly increases understanding.

There exists a visible tendency to overinterpret the statistical significance and importance of *P*-values. It needs to be remembered that statistical significance does not imply clinical relevance, no matter which significance threshold is used. Thus, even if *P*-values associated with a bicluster in one method are smaller than in the other, it does not necessarily mean that the method is performing better. Similarly, a higher percentage of significantly enriched biclusters does not imply superiority because this number might have been inflated by large overlapping biclusters. It may be useful to perform filtering of the returned results or to compare the results with a random detection scenario.

## Conclusions

Although some very powerful techniques have already been developed for big data, there is still a high demand for scalable methods that can provide interpretable insights. One such technique is biclustering, which looks for local associations in data. Biclustering has previously proven its usefulness, especially in the biomedical sciences.

With the recent progress in the development of highly scalable solutions, biclustering is on a good track to become one of the standards of big data analytics.

## Abbreviations

CE: clustering error; CPU: central processing unit; EBIC: evolutionary search-based biclustering; GPU: graphics processing unit; RNA-seq: RNA sequencing; scRNA-seq: single-cell RNA sequencing; TPU: tensor processing unit.

## Competing interests

The authors declare that they have no competing interests.

## Funding

This work was supported by National Institutes of Health grant LM012601.

## Authors' contributions

Original draft preparation: P.O.; review and editing: K.B. and J.H.M.

## Supplementary Material

giz078_GIGA-D-19-00129_Original_Submission

giz078_GIGA-D-19-00129_Revision_1

giz078_Response_to_Reviewer_Comments_Original_Submission

giz078_Reviewer_1_Report_Original_Submission Paul Horton -- 5/19/2019 Reviewed

giz078_Reviewer_2_Report_Original_Submission Jing Zhao -- 5/23/2019 Reviewed
