# Supplementary material for: Scalable biclustering — the future of big data exploration?
Source: Gigascience. 2019 Jun 28;8(7):giz078. doi: 10.1093/gigascience/giz078 (PMC6598466; doi:10.1093/gigascience/giz078)
Supplement: giz078_GIGA-D-19-00129_Original_Submission [file giz078_giga-d-19-00129_original_submission.pdf]

GigaScience

Scalable biclustering - the future of big data exploration?

--Manuscript Draft--

**Manuscript Number:**	GIGA-D-19-00129
**Full Title:**	Scalable biclustering - the future of big data exploration?
**Article Type:**	Commentary
**Funding Information:**	National Institutes of Health (LM012601) — Dr. Jason H. Moore
**Abstract:**	Biclustering is a technique of discovering local similarities within data. For many years the complexity of the meods and parallelization issues limited its application to big data problems. With development of novel scalable methods, biclustering has finally started to close this gap. In this paper we discuss caveats of biclustering, present its current challenges and guidelines for practitioners. We also try to explain why biclustering may become one of the standards for big data analytics.
**Corresponding Author:**	Patryk Orzechowski<br>University of Pennsylvania<br>UNITED STATES
**Corresponding Author Secondary Information:**	
**Corresponding Author's Institution:**	University of Pennsylvania
**Corresponding Author's Secondary Institution:**	
**First Author:**	Patryk Orzechowski, Ph.D.
**First Author Secondary Information:**	
**Order of Authors:**	Patryk Orzechowski, Ph.D.
	Krzysztof Boryczko, Ph.D.
	Jason H. Moore, Ph.D.
**Order of Authors Secondary Information:**	
**Additional Information:**	
**Question**	**Response**
Are you submitting this manuscript to a special series or article collection?	No
**Experimental design and statistics**<br><br>Full details of the experimental design and statistical methods used should be given in the Methods section, as detailed in our Minimum Standards Reporting Checklist. Information essential to interpreting the data presented should be made available in the figure legends.<br><br>Have you included all the information requested in your manuscript?	Yes

Powered by Editorial Manager® and ProduXion Manager® from Aries Systems Corporation

**Resources**<br><br>A description of all resources used, including antibodies, cell lines, animals and software tools, with enough information to allow them to be uniquely identified, should be included in the Methods section. Authors are strongly encouraged to cite Research Resource Identifiers (RRIDs) for antibodies, model organisms and tools, where possible.<br><br>Have you included the information requested as detailed in our Minimum Standards Reporting Checklist?	Yes
**Availability of data and materials**<br><br>All datasets and code on which the conclusions of the paper rely must be either included in your submission or deposited in publicly available repositories (where available and ethically appropriate), referencing such data using a unique identifier in the references and in the "Availability of Data and Materials" section of your manuscript.<br><br>Have you have met the above requirement as detailed in our Minimum Standards Reporting Checklist?	Yes

Powered by Editorial Manager® and ProduXion Manager® from Aries Systems Corporation

Manuscript Click here to download Manuscript main.tex

OXFORD (GIGA)^n SCIENCE

GigaScience, 2017, 1–4

doi: xx.xxxx/xxxx
Manuscript in Preparation
Commentary

COMMENTARY

Scalable biclustering – the future of big data exploration?

Patryk Orzechowski[1,2*], Krzysztof Boryczko[3] and Jason H. Moore[1*]

[1]Institute for Biomedical Informatics, University of Pennsylvania, 3700 Hamilton Walk, Philadelphia, PA 19104, USA and [2]Department of Automatics and Robotics, AGH University of Science and Technology, al. A. Mickiewicza 30, Kraków, 30-059, Poland and [3]Department of Computer Science, AGH University of Science and Technology, al. A. Mickiewicza 30, Kraków, 30-059, Poland

*Corresponding author: patryk.orzechowski@gmail.com

Abstract

Biclustering is a technique of discovering local similarities within data. For many years the complexity of the methods and parallelization issues limited its application to big data problems. With development of novel scalable methods, biclustering has finally started to close this gap. In this paper we discuss caveats of biclustering, present its current challenges and guidelines for practitioners. We also try to explain why biclustering may become one of the standards for big data analytics.

Key words: biclustering, co-clustering, data mining, big data, parallel algorithms, interpretability

Background

The volume of data is rapidly growing, especially in the biomedical domain. In recent years multiple scientific projects large scale data. In *100,000 Genomes Project* [1], one hundred thousand of whole genomes from National Health Service patients were sequenced in United Kingdom with focus on rare diseases, infectious diseases and cancer. Similar effort is taken all across the world [2]. One million subjects are expected to participate in a recently launched *All of Us* initiative in United States[3]. Their genetic and health data will be gathered in order to foster collaborative research on delivering precision medicine addressing different lifestyles and a wide range of health conditions.

In the era of big data, information retrieval becomes key. There is an emerging need of developing new tools that could face large amounts of data challenge. The methods are expected not only to be precise, but also scalable and fast, tolerant to noise. The results they provide are expected to be interpretable in order to provide better understanding of underlying structures in the data. Moreover, the tools are required to capture local similarities in the data, which reflect high heterogeneity.

One of the areas of research in which great progress has been made in recent years to address aforementioned big data challenges is biclustering [1, 2, 3]. This analytical technique of data mining, which is also known as subspace clustering, co-clustering, block clustering, or two-mode clustering, has already become an essential tool for gene expression analysis, as it is capable of capturing similar gene expression profiles under different subset of experimental conditions [4]. It is not without reason that biclustering has found hundreds of applications in bioinformatics and, as a result, there has been a call for increased use of this approach [5]. The era of biclustering big data has begun.

What is biclustering?

There are multiple formulations of biclustering problem, so as multiple challenges. Generally, biclustering is a task of identifying a single or many biclusters, where each of the biclusters is a subset of rows with similar behavior across a subset of columns (or vice-versa) and meets certain criteria of homogeneity [6]. Biclustering could also be viewed from different angles: as detection of sub-matrices, cliques in a bipartite graph, or communities.

Although biclustering is generally considered an unsuper-

Compiled on: April 16, 2019.
Draft manuscript prepared by the author.

Key Points

- Biclustering is a powerful data mining technique aimed at detecting local associations in data.
- There have been multiple successful applications of biclustering in bioinformatics and beyond.
- One of the major advantages of biclustering is interpretability of the results.
- Scalability remains one of the major challenges for algorithms development.
- Recently developed biclustering methods allow to efficiently analyze large scale data.
- Clustering Error (CE) should become a more popular measure for biclustering algorithms performance.
- The resulting set of biclusters should be ordered according to their relevance.

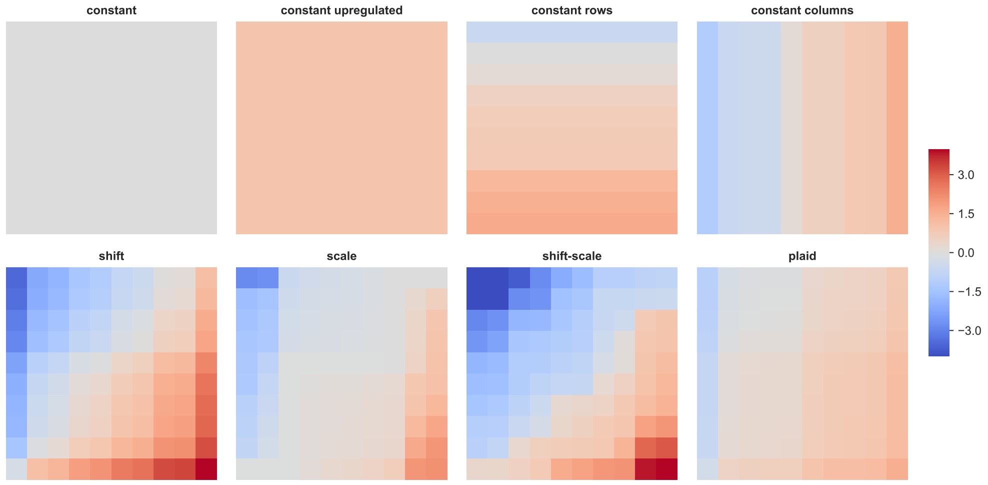

Figure 1. Different patterns in biclustering. The original patterns were sorted first by rows and secondly by columns for visualization purposes. Notice that all the patterns are order-preserving.

vised machine learning technique multiple semi-supervised or supervised approaches have been proposed, which are based on related concepts. Biclustering is closely related to fuzzy clustering, frequent itemset mining, as well as learning classifiers systems. Depending on the field of application, the data for the algorithms could be numerical (binary, discrete or continuous), categorical or ordinal. The methods may attempt to detect a single bicluster, exclusive biclusters (their rows, columns, or both may belong to no more than a single bicluster), disjoint biclusters (i.e. non-overlapping, e.g. checkerboard), inclusive biclusters (the only overlap between biclusters could be inclusion), or arbitrary positioned biclusters. The patterns to be detected can also vary, starting from classical biclustering problems (constant values, upregulated values, constant values in rows, constant values in columns, shift patterns, scale patterns, shift-scale patterns, plaid patterns, order preserving – coherent evolutions) [7]. The most popular data patterns for biclustering generated using BiBench [4] are presented in Figure 1.

Common myths about biclustering

Let us demystify some common views on biclustering.

The only application of biclustering is gene expression. This claim is too narrow. Biclustering has been successfully applied to hundreds of problems in biological and biomedical domain [5]. The techniques have been also been successfully used in text mining, recommendation systems, marketing, economy (e.g. market segmentation), analysis of sport data and multiple other domains [1]. Certainly, gene expression data could be considered a common playground, as this is where the most important discoveries are made.

Is biclustering a local version of clustering? This is correct. Biclustering looks for local similarities within the data, what could be seen as performing clustering locally.

Is biclustering the same as two way clustering? Not necessarily. Although some of the first biclustering methods used to cluster first by rows, then by columns (or the other way around), the field has progressed far since its emergence. Usually biclustering techniques use information from both rows and columns at the same time, or alternately from rows and columns to progress.

Biclustering = feature selection + clustering? Although it seems likely, this is not true. Usually different features contribute to different biclusters. There are some common aspects though, as for each bicluster certain features are selected. The closest answer is that biclustering borrows from both techniques, but is certainly not combination of both.

Is biclustering a dimensionality reduction technique? The answer is no, but biclustering can be used as a technique that reduces dimensionality, as it finds patterns with subsets of rows and columns with very similar characteristics.

Biclustering is neither generative, nor predictive. True. Biclustering algorithms are usually expected to retrieve existing (but hidden) information and thus provide insight into data. The methods aren't intended not to generate the data, nor to make predictions. Biclustering methods are intended to locate spe-

cific patterns, which they were designed for.

Biclustering is much more complex than deep learning. The majority of problems in biclustering are considered NP-complete [6]. To better visualize the complexity of usual biclustering problem, let us consider a task of detecting an object on an image. Biclustering task would be formulated as finding the same object, but in the image with randomly shuffled rows and randomly shuffled columns. The assumption that neighboring rows or columns belong to the same object, or that two related objects are next to each other (e.g. words creating context) greatly simplifies the problem.

Biclustering and big data

Biclustering field has largely evolved since its first application to gene expression in 2000. Modern methods take advantage of parallel computation, or map-reduce paradigm. The popular environments for launching large scale biclustering analyses are becoming Hadoop [5], Apache Spark [6] and massively parallel systems with multiple GPUs [4].

Recently, a very accurate and scalable method for biclustering big data called EBIC was proposed for multi-GPU environment [8, 2]. This open source method [7] manages to detect multiple patterns in the data and scales very well for large datasets. Its latest release allows to omit missing values, what makes the method applicable to RNA-seq and single cell RNA-seq (scRNA-seq) data. Another biclustering method called Progressive Bicluster Extension (PBE) applied to big has also helped to helped to identify novel human microRNA regulatory modules [3].

Challenges of biclustering

We would like to summarize the major challenges that biclustering currently faces.

Scalability It is crucial to understand where parallelization in the method may be exploited [4], or - as we suggest - even start developing the method by acknowledging the hardware limitations. This is exactly the way how EBIC was developed. The method was designed in compliance with GPU memory limitations and programming constraints from the very beginning.

What size of the biclusters? This is an open-ended question which patterns (local or global, narrow or wide?) are more important, and the answer probably depends on the application. Big data is definitely not helping here and the task becomes sometimes looking for a needle in a haystack.

How many biclusters? Usually biclustering methods yield either requested number, or up to 100 of biclusters, but some algorithms may return even millions of patterns. We strongly recommend ranking all biclusters and reporting the biclusters with the highest relevance first.

How to measure the performance? The most established measures in the field, called recovery and relevance, are based on Jaccard index, and were shown to be inadequate for objective assessment of performance of biclustering methods. Horta and Campello reviewed different measures for biclustering and presented their desirable properties [9]. Biclustering measures should increasingly penalize noisy entries or elements not found in both biclusterings. Not covering all solutions, reporting elements not belonging to a specific bicluster, as well as covering the same elements multiple times should be penalized as well. Repetitively reporting the same reference bicluster is also not desirable. Finally, the measure should be symmetric and return score equal to 1 for a perfect fit. Although the authors reported two measures that have the desired properties, only Clustering Error (CE) [10] doesn't penalize heterogeneous patterns, which are very common in genomics. Thus, we believe that CE should be considered as the most objective measure of performance of biclustering methods.

Interpretability is the key

One of the major advantages of biclustering over many other methods (e.g. feature selection) is interpretability of the results. Biclusters are usually very easy to interpret, as they extract very specific patterns (e.g. all the values of a bicluster are constant, or each rows within the bicluster are correlated). Interpretability greatly helps understanding.

For synthetic datasets, it is very useful to realize what types of patterns can be detected with a given methods. In biomedical domain, interpretation is performed using expert knowledge. Gene set enrichment analysis, or pathway analysis are common techniques of validation. Additional caution needs to be taken when interpreting the performance of the methods. For example, below a certain very small threshold p-values become numerically meaningless. Similarly, higher percentage of significantly enriched biclusters doesn't necessarily mean that the method is performing better, as large overlapping biclusters might have artificially inflated the percentage.

Conclusions

Although some very powerful techniques have already been developed for big data, there is still a very high demand for scalable methods that can provide interpretable insights. One such technique is biclustering, which looks for local associations in data. Biclustering has previously proven its usefulness, especially in biomedical sciences.

With recent progress in development of highly scalable solutions, biclustering is on the perfect way of becoming one of the standards of big data analytics.

Competing interests

The authors declare that they have no competing interests.

Funding

This work was supported by NIH grant LM012601.

Author contribution

Original draft preparation: P.O.; review and editing: K.B. and J.H.M..

References

1. Kasim A, Shkedy Z, Kaiser S, Hochreiter S, Talloen W. Applied biclustering methods for big and high-dimensional data using R. CRC Press; 2016.
2. Orzechowski P, Moore JH. EBIC: an open source software for high-dimensional and big data analyses. Bioinformatics 2019;p. btz027.
3. Yoon S, Nguyen HCT, Jo W, Kim J, Chi SM, Park J, et al.

Biclustering analysis of transcriptome big data identifies condition-specific microRNA targets. Nucleic acids research 2019;.
4. Gomez-Vela F, López A, Lagares JA, Baena DS, Barranco CD, García-Torres M, et al. Bioinformatics from a Big Data Perspective: Meeting the Challenge. In: International Conference on Bioinformatics and Biomedical Engineering Springer; 2017. p. 349–359.
5. Xie J, Ma A, Fennell A, Ma Q, Zhao J. It is time to apply biclustering: a comprehensive review of biclustering applications in biological and biomedical data. Briefings in bioinformatics 2018;.
6. Madeira SC, Oliveira AL. Biclustering algorithms for biological data analysis: a survey. IEEE/ACM Transactions on Computational Biology and Bioinformatics (TCBB) 2004;1(1):24–45.
7. Padilha VA, Campello RJ. A systematic comparative evaluation of biclustering techniques. BMC bioinformatics 2017;18(1):55.
8. Orzechowski P, Sipper M, Huang X, Moore JH. EBIC: an evolutionary-based parallel biclustering algorithm for pattern discovery. Bioinformatics 2018 05;34(21):3719–3726.
9. Horta D, Campello RJ. Similarity measures for comparing biclusterings. IEEE/ACM Transactions on Computational Biology and Bioinformatics (TCBB) 2014;11(5):942–954.
10. Patrikainen A, Meila M. Comparing subspace clusterings. IEEE Transactions on Knowledge and Data Engineering 2006;18(7):902–916.

Notes

[1]https://www.genomicsengland.co.uk/about-genomics-england/the-100000-genomes-project/
[2]https://www.clinicalomics.com/topics/biomarkers-topic/biobanking/10-countries-in-100k-genome-club/
[3]https://allofus.nih.gov/
[4]http://tda.gatech.edu/software/bibench-v0.2/
[5]https://hadoop.apache.org/
[6]https://spark.apache.org/
[7]https://github.com/EpistasisLab/ebic

Manuscript Click here to download Manuscript Reference PDF.pdf

GigaScience, 2017, 1–4

doi: xx.xxxx/xxxx
Manuscript in Preparation
Commentary

COMMENTARY

Scalable biclustering – the future of big data exploration?

Patryk Orzechowski[1,2*], Krzysztof Boryczko[3] and Jason H. Moore[1*]

[1]Institute for Biomedical Informatics, University of Pennsylvania, 3700 Hamilton Walk, Philadelphia, PA 19104, USA and [2]Department of Automatics and Robotics, AGH University of Science and Technology, al. A. Mickiewicza 30, Kraków, 30-059, Poland and [3]Department of Computer Science, AGH University of Science and Technology, al. A. Mickiewicza 30, Kraków, 30-059, Poland

*Corresponding authors: patryk.orzechowski@gmail.com; jhmoore@upenn.edu

Abstract

Biclustering is a technique of discovering local similarities within data. For many years the complexity of the methods and parallelization issues limited its application to big data problems. With development of novel scalable methods, biclustering has finally started to close this gap. In this paper we discuss caveats of biclustering, present its current challenges and guidelines for practitioners. We also try to explain why biclustering may become one of the standards for big data analytics.

Key words: biclustering, co-clustering, data mining, big data, parallel algorithms, interpretability

Background

The volume of data is rapidly growing, especially in the biomedical domain. In recent years multiple scientific projects large scale data. In *100,000 Genomes Project* [1], one hundred thousand of whole genomes from National Health Service patients were sequenced in United Kingdom with focus on rare diseases, infectious diseases and cancer. Similar effort is taken all across the world [2]. One million subjects are expected to participate in a recently launched *All of Us* initiative in United States[3]. Their genetic and health data will be gathered in order to foster collaborative research on delivering precision medicine addressing different lifestyles and a wide range of health conditions.

In the era of big data, information retrieval becomes key. There is an emerging need of developing new tools that could face large amounts of data challenge. The methods are expected not only to be precise, but also scalable and fast, tolerant to noise. The results they provide are expected to be interpretable in order to provide better understanding of underlying structures in the data. Moreover, the tools are required to capture local similarities in the data, which reflect high heterogeneity.

One of the areas of research in which great progress has been made in recent years to address aforementioned big data challenges is biclustering [1, 2, 3]. This analytical technique of data mining, which is also known as subspace clustering, co-clustering, block clustering, or two-mode clustering, has already become an essential tool for gene expression analysis, as it is capable of capturing similar gene expression profiles under different subset of experimental conditions [4]. It is not without reason that biclustering has found hundreds of applications in bioinformatics and, as a result, there has been a call for increased use of this approach [5]. The era of biclustering big data has begun.

What is biclustering?

There are multiple formulations of biclustering problem, so as multiple challenges. Generally, biclustering is a task of identifying a single or many biclusters, where each of the biclusters is a subset of rows with similar behavior across a subset of columns (or vice-versa) and meets certain criteria of homogeneity [6]. Biclustering could also be viewed from different angles: as detection of sub-matrices, cliques in a bipartite graph, or communities.

Although biclustering is generally considered an unsuper-

Compiled on: April 16, 2019.
Draft manuscript prepared by the author.

Key Points

- Biclustering is a powerful data mining technique aimed at detecting local associations in data.
- There have been multiple successful applications of biclustering in bioinformatics and beyond.
- One of the major advantages of biclustering is interpretability of the results.
- Scalability remains one of the major challenges for algorithms development.
- Recently developed biclustering methods allow to efficiently analyze large scale data.
- Clustering Error (CE) should become a more popular measure for biclustering algorithms performance.
- The resulting set of biclusters should be ordered according to their relevance.

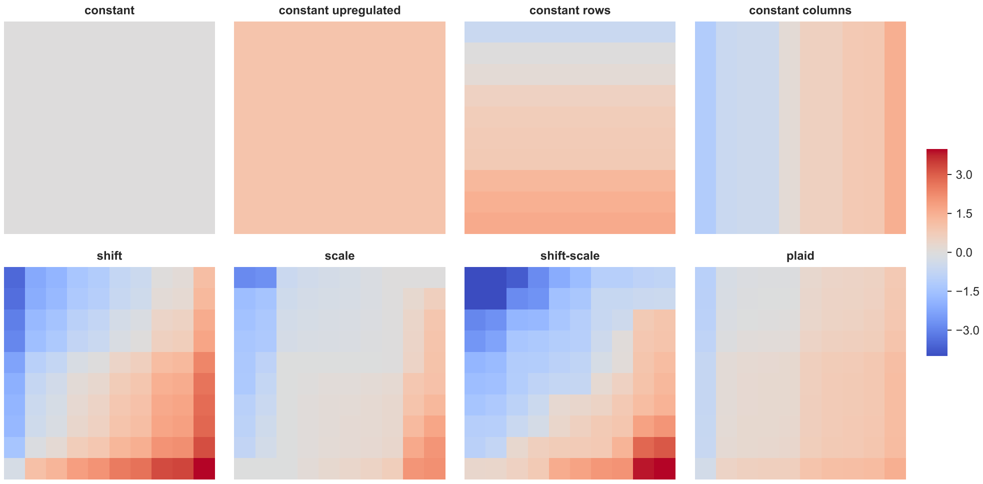

Figure 1. Different patterns in biclustering. The original patterns were sorted first by rows and secondly by columns for visualization purposes. Notice that all the patterns are order-preserving.

vised machine learning technique multiple semi-supervised or supervised approaches have been proposed, which are based on related concepts. Biclustering is closely related to fuzzy clustering, frequent itemset mining, as well as learning classifiers systems. Depending on the field of application, the data for the algorithms could be numerical (binary, discrete or continuous), categorical or ordinal. The methods may attempt to detect a single bicluster, exclusive biclusters (their rows, columns, or both may belong to no more than a single bicluster), disjoint biclusters (i.e. non-overlapping, e.g. checkerboard), inclusive biclusters (the only overlap between biclusters could be inclusion), or arbitrary positioned biclusters. The patterns to be detected can also vary, starting from classical biclustering problems (constant values, upregulated values, constant values in rows, constant values in columns, shift patterns, scale patterns, shift-scale patterns, plaid patterns, order preserving – coherent evolutions) [7]. The most popular data patterns for biclustering generated using BiBench [4] are presented in Figure 1.

Common myths about biclustering

Let us demystify some common views on biclustering.

The only application of biclustering is gene expression. This claim is too narrow. Biclustering has been successfully applied to hundreds of problems in biological and biomedical domain [5]. The techniques have been also been successfully used in text mining, recommendation systems, marketing, economy (e.g. market segmentation), analysis of sport data and multiple other domains [1]. Certainly, gene expression data could be considered a common playground, as this is where the most important discoveries are made.

Is biclustering a local version of clustering? This is correct. Biclustering looks for local similarities within the data, what could be seen as performing clustering locally.

Is biclustering the same as two way clustering? Not necessarily. Although some of the first biclustering methods used to cluster first by rows, then by columns (or the other way around), the field has progressed far since its emergence. Usually biclustering techniques use information from both rows and columns at the same time, or alternately from rows and columns to progress.

Biclustering = feature selection + clustering? Although it seems likely, this is not true. Usually different features contribute to different biclusters. There are some common aspects though, as for each bicluster certain features are selected. The closest answer is that biclustering borrows from both techniques, but is certainly not combination of both.

Is biclustering a dimensionality reduction technique? The answer is no, but biclustering can be used as a technique that reduces dimensionality, as it finds patterns with subsets of rows and columns with very similar characteristics.

Biclustering is neither generative, nor predictive. True. Biclustering algorithms are usually expected to retrieve existing (but hidden) information and thus provide insight into data. The methods aren't intended not to generate the data, nor to make predictions. Biclustering methods are intended to locate spe-

cific patterns, which they were designed for.

Biclustering is much more complex than deep learning. The majority of problems in biclustering are considered NP-complete [6]. To better visualize the complexity of usual biclustering problem, let us consider a task of detecting an object on an image. Biclustering task would be formulated as finding the same object, but in the image with randomly shuffled rows and randomly shuffled columns. The assumption that neighboring rows or columns belong to the same object, or that two related objects are next to each other (e.g. words creating context) greatly simplifies the problem.

Biclustering and big data

Biclustering field has largely evolved since its first application to gene expression in 2000. Modern methods take advantage of parallel computation, or map-reduce paradigm. The popular environments for launching large scale biclustering analyses are becoming Hadoop [5], Apache Spark [6] and massively parallel systems with multiple GPUs [4].

Recently, a very accurate and scalable method for biclustering big data called EBIC was proposed for multi-GPU environment [8, 2]. This open source method [7] manages to detect multiple patterns in the data and scales very well for large datasets. Its latest release allows to omit missing values, what makes the method applicable to RNA-seq and single cell RNA-seq (scRNA-seq) data. Another biclustering method called Progressive Bicluster Extension (PBE) applied to big has also helped to helped to identify novel human microRNA regulatory modules [3].

Challenges of biclustering

We would like to summarize the major challenges that biclustering currently faces.

Scalability It is crucial to understand where parallelization in the method may be exploited [4], or - as we suggest - even start developing the method by acknowledging the hardware limitations. This is exactly the way how EBIC was developed. The method was designed in compliance with GPU memory limitations and programming constraints from the very beginning.

What size of the biclusters? This is an open-ended question which patterns (local or global, narrow or wide?) are more important, and the answer probably depends on the application. Big data is definitely not helping here and the task becomes sometimes looking for a needle in a haystack.

How many biclusters? Usually biclustering methods yield either requested number, or up to 100 of biclusters, but some algorithms may return even millions of patterns. We strongly recommend ranking all biclusters and reporting the biclusters with the highest relevance first.

How to measure the performance? The most established measures in the field, called recovery and relevance, are based on Jaccard index, and were shown to be inadequate for objective assessment of performance of biclustering methods. Horta and Campello reviewed different measures for biclustering and presented their desirable properties [9]. Biclustering measures should increasingly penalize noisy entries or elements not found in both biclusterings. Not covering all solutions, reporting elements not belonging to a specific bicluster, as well as covering the same elements multiple times should be penalized as well. Repetitively reporting the same reference bicluster is also not desirable. Finally, the measure should be symmetric and return score equal to 1 for a perfect fit. Although the authors reported two measures that have the desired properties, only Clustering Error (CE) [10] doesn't penalize heterogeneous patterns, which are very common in genomics. Thus, we believe that CE should be considered as the most objective measure of performance of biclustering methods.

Interpretability is the key

One of the major advantages of biclustering over many other methods (e.g. feature selection) is interpretability of the results. Biclusters are usually very easy to interpret, as they extract very specific patterns (e.g. all the values of a bicluster are constant, or each rows within the bicluster are correlated). Interpretability greatly helps understanding.

For synthetic datasets, it is very useful to realize what types of patterns can be detected with a given methods. In biomedical domain, interpretation is performed using expert knowledge. Gene set enrichment analysis, or pathway analysis are common techniques of validation. Additional caution needs to be taken when interpreting the performance of the methods. For example, below a certain very small threshold p-values become numerically meaningless. Similarly, higher percentage of significantly enriched biclusters doesn't necessarily mean that the method is performing better, as large overlapping biclusters might have artificially inflated the percentage.

Conclusions

Although some very powerful techniques have already been developed for big data, there is still a very high demand for scalable methods that can provide interpretable insights. One such technique is biclustering, which looks for local associations in data. Biclustering has previously proven its usefulness, especially in biomedical sciences.

With recent progress in development of highly scalable solutions, biclustering is on the perfect way of becoming one of the standards of big data analytics.

Competing interests

The authors declare that they have no competing interests.

Funding

This work was supported by NIH grant LM012601.

Author contribution

Original draft preparation: P.O.; review and editing: K.B. and J.H.M..

References

1. Kasim A, Shkedy Z, Kaiser S, Hochreiter S, Talloen W. Applied biclustering methods for big and high-dimensional data using R. CRC Press; 2016.
2. Orzechowski P, Moore JH. EBIC: an open source software for high-dimensional and big data analyses. Bioinformatics 2019;p. btz027.
3. Yoon S, Nguyen HCT, Jo W, Kim J, Chi SM, Park J, et al.

Biclustering analysis of transcriptome big data identifies condition-specific microRNA targets. Nucleic acids research 2019;.
4. Gomez-Vela F, López A, Lagares JA, Baena DS, Barranco CD, García-Torres M, et al. Bioinformatics from a Big Data Perspective: Meeting the Challenge. In: International Conference on Bioinformatics and Biomedical Engineering Springer; 2017. p. 349–359.
5. Xie J, Ma A, Fennell A, Ma Q, Zhao J. It is time to apply biclustering: a comprehensive review of biclustering applications in biological and biomedical data. Briefings in bioinformatics 2018;.
6. Madeira SC, Oliveira AL. Biclustering algorithms for biological data analysis: a survey. IEEE/ACM Transactions on Computational Biology and Bioinformatics (TCBB) 2004;1(1):24–45.
7. Padilha VA, Campello RJ. A systematic comparative evaluation of biclustering techniques. BMC bioinformatics 2017;18(1):55.
8. Orzechowski P, Sipper M, Huang X, Moore JH. EBIC: an evolutionary-based parallel biclustering algorithm for pattern discovery. Bioinformatics 2018 05;34(21):3719–3726.
9. Horta D, Campello RJ. Similarity measures for comparing biclusterings. IEEE/ACM Transactions on Computational Biology and Bioinformatics (TCBB) 2014;11(5):942–954.
10. Patrikainen A, Meila M. Comparing subspace clusterings. IEEE Transactions on Knowledge and Data Engineering 2006;18(7):902–916.

Notes

[1]https://www.genomicsengland.co.uk/about-genomics-england/the-100000-genomes-project/
[2]https://www.clinicalomics.com/topics/biomarkers-topic/biobanking/10-countries-in-100k-genome-club/
[3]https://allofus.nih.gov/
[4]http://tda.gatech.edu/software/bibench-v0.2/
[5]https://hadoop.apache.org/
[6]https://spark.apache.org/
[7]https://github.com/EpistasisLab/ebic

Cover letter
Click here to download Personal Cover submission-letter-gigascience-2.pdf

Perelman School of Medicine
University of Pennsylvania

Institute for Biomedical Informatics

Jason H. Moore, PhD
Edward Rose Professor of Informatics
Director, Institute for Biomedical Informatics
Director, Division of Informatics
Senior Associate Dean for Informatics

April 15, 2019

Dear Editors,

Following our previous correspondence, attached please find our commentary on scalable biclustering. In the paper "Scalable biclustering – the future of big data exploration?" we focus on the recent advances in biclustering. We also share our previous experience in developing scalable methods and discuss major challenges that biclustering faces from big data perspective.

Biclusering is a well-established unsupervised machine learning technique which detects subsets of rows and subsets of columns that are associated with each other. For almost 20 years, since its first application to genomic data, hundreds of different approaches emerged. Very few approaches however focused on data scalability.

With increasing volume of biomedical data, high heterogeneity, presence of noise and outliers it is crucial to foster collaborative research on developing next-generation biclustering techniques that are both accurate and scalable. One of the recent advances in the field is development of Evolutionary-search based Biclustering (EBIC), a multi-GPU parallel biclustering method that outperformed multiple approaches in terms of accuracy. The method was further improved to work on big data in 2019.

Thank you for your consideration.

Sincerely,

Jason H. Moore, Ph.D.
Director, Penn Institute for Biomedical Informatics

D202 Richards Building, 3700 Hamilton Walk • Philadelphia, PA 19104-6116 • Tel: (215) 573-4411 • Fax: (215) 573-3111
Email: jhmoore@upenn.edu
