# Supplementary material for: Scalable biclustering — the future of big data exploration?
Source: Gigascience. 2019 Jun 28;8(7):giz078. doi: 10.1093/gigascience/giz078 (PMC6598466; doi:10.1093/gigascience/giz078)
Supplement: giz078_GIGA-D-19-00129_Revision_1 [file giz078_giga-d-19-00129_revision_1.pdf]

GigaScience

Scalable biclustering - the future of big data exploration?

--Manuscript Draft--

**Manuscript Number:**	GIGA-D-19-00129R1
**Full Title:**	Scalable biclustering - the future of big data exploration?
**Article Type:**	Commentary
**Funding Information:**	National Institutes of Health (LM012601) — Dr. Jason H. Moore
**Abstract:**	Biclustering is a technique of discovering local similarities within data. For many years the complexity of the methods and parallelization issues limited its application to big data problems. With development of novel scalable methods, biclustering has finally started to close this gap. In this paper we discuss caveats of biclustering, present its current challenges and guidelines for practitioners. We also try to explain why biclustering may soon become one of the standards for big data analytics.
**Corresponding Author:**	Patryk Orzechowski<br>University of Pennsylvania<br>UNITED STATES
**Corresponding Author Secondary Information:**	
**Corresponding Author's Institution:**	University of Pennsylvania
**Corresponding Author's Secondary Institution:**	
**First Author:**	Patryk Orzechowski, Ph.D.
**First Author Secondary Information:**	
**Order of Authors:**	Patryk Orzechowski, Ph.D.
	Krzysztof Boryczko, Ph.D.
	Jason H. Moore, Ph.D.
**Order of Authors Secondary Information:**	
**Response to Reviewers:**	Response to Reviewer #1<br>We thank the Reviewer for a positive feedback. We completely agree that many aspects in the manuscript were either barely mentioned or not fully covered. The main reason for this is the limitations in the size and number of the references of the commentary.<br><br>To address the Reviewer's comments we have expanded a "What is biclustering?" section by providing a formal definition of biclustering. We have also reorganized a paragraph (former paragraph in common myths section) into a separate section "What is the application of biclustering?". The section was expanded by mentioning several biomedical case studies. Additional information with more detailed coverage of use cases could be found in one of the references.<br><br>Additionally, we have rephrased and provided additional explanation for the paragraph on results interpretation.<br><br>Response to Reviewer #2<br><br>We would like to thank the Reviewer for reviewing our manuscript.<br><br>We have added clarification on scalable biclustering and created "Scalable biclustering" section.

Powered by Editorial Manager® and ProduXion Manager® from Aries Systems Corporation

	A more detailed description was added in the figure caption.<br><br>We have clarified that algorithm developers should pay more attention in providing the users parameters that could adjust the method to specific purposes. In our opinion no recommendation could be given on the size and number of biclusters, as this is problem-specific. For example, if the goal of the study is to find cohorts of patients, detection of large biclusters is encouraged, as the goal is to cover as many patients with a common characteristics as possible. However, if the goal is detection of gene regulatory network, large biclusters often contain lot of noise and such information extracted may not be very informative.<br><br>We would also like to emphasize that only the most relevant studies were referenced in the paper due to the limitation of the format of the paper.
Additional Information:	
Question	Response
Are you submitting this manuscript to a special series or article collection?	No
**Experimental design and statistics**<br><br>Full details of the experimental design and statistical methods used should be given in the Methods section, as detailed in our Minimum Standards Reporting Checklist. Information essential to interpreting the data presented should be made available in the figure legends.<br><br>Have you included all the information requested in your manuscript?	Yes
**Resources**<br><br>A description of all resources used, including antibodies, cell lines, animals and software tools, with enough information to allow them to be uniquely identified, should be included in the Methods section. Authors are strongly encouraged to cite Research Resource Identifiers (RRIDs) for antibodies, model organisms and tools, where possible.<br><br>Have you included the information requested as detailed in our Minimum Standards Reporting Checklist?	Yes
**Availability of data and materials**	Yes

All datasets and code on which the conclusions of the paper rely must be either included in your submission or deposited in publicly available repositories (where available and ethically appropriate), referencing such data using a unique identifier in the references and in the “Availability of Data and Materials” section of your manuscript.<br><br>Have you have met the above requirement as detailed in our Minimum Standards Reporting Checklist?	

Powered by Editorial Manager® and ProduXion Manager® from Aries Systems Corporation

Manuscript
Click here to access/download;Manuscript;main.tex
Click here to view linked References

GigaScience, 2017, 1–4

doi: xx.xxxx/xxxx
Manuscript in Preparation
Commentary

COMMENTARY

Scalable biclustering – the future of big data exploration?

Patryk Orzechowski[1,2*], Krzysztof Boryczko[3] and Jason H. Moore[1*]

[1]Institute for Biomedical Informatics, University of Pennsylvania, 3700 Hamilton Walk, Philadelphia, PA 19104, USA and [2]Department of Automatics and Robotics, AGH University of Science and Technology, al. A. Mickiewicza 30, Kraków, 30-059, Poland and [3]Department of Computer Science, AGH University of Science and Technology, al. A. Mickiewicza 30, Kraków, 30-059, Poland

[*]Corresponding author: patryk.orzechowski@gmail.com

Abstract

Biclustering is a technique of discovering local similarities within data. For many years the complexity of the methods and parallelization issues limited its application to big data problems. With development of novel scalable methods, biclustering has finally started to close this gap. In this paper we discuss caveats of biclustering, present its current challenges and guidelines for practitioners. We also try to explain why biclustering may soon become one of the standards for big data analytics.

Key words: biclustering, co-clustering, data mining, big data, parallel algorithms, disease subtype identification, biomarker detection, gene-drug interaction, precision medicine

Background

The volume of data is rapidly growing, especially in the biomedical domain. In recent years multiple scientific projects large scale data. In *100,000 Genomes Project* [1], one hundred thousand of whole genomes from National Health Service patients were sequenced in the United Kingdom with focus on rare diseases, infectious diseases, and cancer. A similar effort is taken all across the world [2]. One million subjects are expected to participate in a recently launched *All of Us* initiative in United States[3]. Their genetic and health data will be gathered in order to foster collaborative research on delivering precision medicine addressing different lifestyles and a wide range of health conditions.

In the era of big data, information retrieval becomes key. There is an emerging need for developing tools that could face the challenge of large amounts of data. The methods are expected not only to be precise but also tolerant to noise, scalable, and fast. The results are expected to be interpretable in order to provide a better understanding of underlying structures in the data. Moreover, the tools are required to capture local similarities in the data, which reflect high heterogeneity.

One of the areas of research in which great progress has been made in recent years to address the aforementioned big data challenges is biclustering [1, 2, 3, 4]. This analytical technique of data mining, which is also known as subspace clustering, co-clustering, block clustering, or two-mode clustering, has already become an essential tool for gene expression analysis, as it is capable of capturing similar gene expression profiles under different subsets of experimental conditions [5]. It is not without reason that biclustering has found hundreds of applications in bioinformatics and, as a result, there has been a call for increased use of this approach [6]. The era of biclustering big data has begun.

What is biclustering?

There are multiple formulations of biclustering problem, so as multiple challenges. Generally, biclustering is a task of identifying a single or many biclusters, where each of the biclusters is a subset of rows with similar behavior across a sub-

Compiled on: June 7, 2019.
Draft manuscript prepared by the author.

Key Points

- Biclustering is a powerful data mining technique aimed at detecting local associations in data.
- There have been multiple successful applications of biclustering in bioinformatics and beyond.
- One of the major advantages of biclustering is interpretability of the results.
- Scalability remains one of the major challenges for algorithms development.
- Recently developed biclustering methods allow to efficiently analyze large scale data.
- Clustering Error (CE) should become a more popular measure for biclustering algorithms performance.
- Ranking biclusters allows focusing on the most relevant biclusters first.

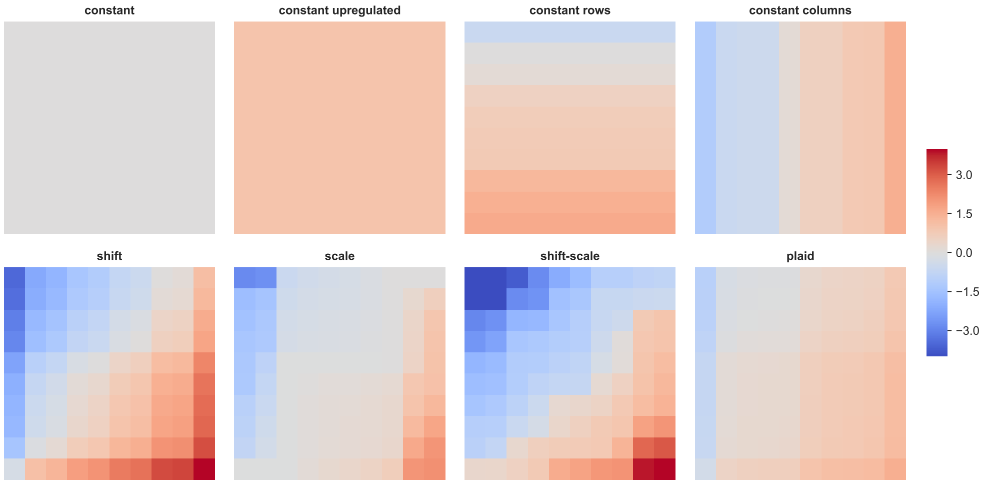

Figure 1. Different patterns in biclustering. The original patterns were sorted first by rows and secondly by columns for visualization purposes. Biclusters with **constant** or **upregulated** pattern have all values exact. **Constant rows/columns** patterns are characterized by the same value across all columns/rows of the bicluster. The values between rows/columns may differ. In a bicluster with **shift** pattern the contribution of a given row is added to the contribution of a given column, whilst in case of **scale** pattern contribution of a row is multiplied. In a **shift-scale** pattern each row contributes twice - by a factor which is multiplied by a column contribution and by an additive further shifting the values. In **plaid** patterns, the data is modeled as a sum of multiple layers. Notice that all the patterns could be considered order-preserving.

set of columns (or vice-versa). For a dataset $A = [a_{ij}]_{m\times n}$ with rows denoted as $X = (x_1, x_2, \ldots, x_m)$, and columns as $Y = (y_1, y_2, \ldots, y_n)$, biclustering is an identification of a single or a series of p biclusters $B_k = (I_k, J_k)$, where $k = 1, 2, \ldots, p$, and $I_k \subseteq X$ are the rows of k-th bicluster, and $J_k \subseteq Y$ are the columns of the k-th bicluster, where each of the biclusters meets some homogeneity criteria [7]. Biclustering could also be viewed from different angles: as detection of sub-matrices, cliques in a bipartite graph, or communities.

Although biclustering is generally considered an unsupervised machine learning technique, multiple semi-supervised or supervised approaches have been proposed which are based on related concepts. Biclustering is closely related to fuzzy clustering, frequent itemset mining, as well as learning classifiers systems. Depending on the field of application, the data for the algorithms could be numerical (binary, discrete or continuous), categorical or ordinal. The methods may attempt to detect a single bicluster, exclusive biclusters (their rows, columns, or both may belong to no more than a single bicluster), disjoint biclusters (i.e. non-overlapping, e.g. checkerboard), inclusive biclusters (the only overlap between biclusters could be inclusion), or arbitrary positioned biclusters. The patterns to be detected can also vary, starting from classical biclustering problems (constant values, upregulated values, constant values in rows, constant values in columns, shift patterns, scale patterns, shift-scale patterns, plaid patterns, order-preserving - coherent evolutions) [8]. Some of the most popular data patterns for biclustering generated using BiBench [4] are presented in Figure 1.

What is the application of biclustering?

Biclustering has been successfully applied to hundreds of problems in the biological and biomedical domain and has supported detection of functional annotations (e.g. gene regulatory pathways) as well as biological interactions (e.g. transcriptional networks), the discovery of drugs and biomarkers, identification of subtypes of diseases, and analyzing responses to treatments [6]. A biclustering method has also helped to identify novel human microRNA regulatory modules [2]. Biclustering techniques have been also been successfully used in graph analysis, text mining, recommendation systems, marketing, economy (e.g. market segmentation), analysis of sports data and multiple other domains [1]. Among multiple domains, gene expression data is commonly considered a real data playground for measuring the performance of biclustering methods.

Common myths about biclustering

Let us demystify some common views on biclustering.

Is biclustering a local equivalent of clustering? This is correct. While clustering looks for global similarities within data and takes into consideration all data dimensions, biclustering cap-

tures patterns hidden locally and uses only some of the data dimensions.

Is biclustering the same as two-way clustering? Not necessarily. Although some of the first biclustering methods used to cluster first by rows, then by columns (or the other way around), the field has progressed far since its emergence. Usually, biclustering techniques either use information from both rows and columns at the same time, or alternately from rows and columns to progress.

Biclustering = feature selection + clustering? Although it seems likely, this is not true. Usually different features contribute to different biclusters. There are some common aspects though, as for each bicluster certain features are selected. The closest answer is that biclustering borrows from both techniques, but is certainly not a combination of both.

Is biclustering a dimensionality reduction technique? The answer is no, but biclustering can be used as a technique that reduces dimensionality, as it finds patterns with subsets of rows and columns with very similar characteristics.

Biclustering is neither generative, nor predictive. True. Biclustering algorithms are usually expected to retrieve existing (but hidden) information and thus provide insight into data. The methods aren't intended to generate the data, nor to make predictions. Biclustering methods are intended to locate specific patterns, which they were designed for.

Biclustering is much more complex than deep learning. The majority of problems in biclustering are considered NP-complete [7]. To better visualize the complexity of usual biclustering problem, let us consider a task of detecting an object on an image. Biclustering task would be formulated as finding the same object, but in the image with randomly shuffled rows and randomly shuffled columns. The assumption that neighboring rows or columns belong to the same object, or that two related objects are next to each other (e.g. words creating context) greatly simplifies the problem and makes deep learning methods much more efficient for image analysis, or natural language processing tasks.

Biclustering and big data

Biclustering field has largely evolved since its first application to gene expression in 2000. Modern methods take advantage of parallel computation or map-reduce paradigm. The popular environments for launching large scale biclustering analyses are becoming Hadoop [5], Apache Spark [6] and massively parallel systems with multiple GPUs [5].

Recently, a very accurate and scalable method for biclustering big data called EBIC was proposed for multi-GPU environment [3, 4]. This open source method [7] manages to detect multiple patterns in the data and scales very well for large datasets. Its latest release allows to omit missing values, which makes the method applicable to RNA-seq and single cell RNA-seq (scRNA-seq) data.

Scalable biclustering

With continuously increasing sizes of the data many traditional biclustering approaches struggle to analyze the data within a reasonable time frame. Large volume of the data, high complexity of the problem, as well as poor memory management of some of the implementations make a lot of approaches not feasible to handle big data problems. Thus, one of the main focuses of future algorithms design is focusing on their scalability. By *scalability* we understand the ability of the method to handle increasing sizes of the data with additional resources (e.g. CPUs, GPUs or TPUs) in a reasonable time.

Considering the algorithm design, it is crucial to understand where and how parallelization in the method may be exploited to provide speedup, so desirable for analyzing large datasets [5]. Alternatively, the development of a new method could start with understanding hardware limitations. From our experience, the second option is more extendable (e.g. EBIC was designed from scratch in compliance with GPU memory limitations and programming constraints).

Challenges of biclustering

Apart from the ability to analyze big data, we would like to summarize some of the major challenges that biclustering currently faces.

What size of the biclusters? This is an open-ended question what sizes of the patterns (local or global? narrow or wide?) are more important, and the answer probably depends on a specific domain of application. Big data is definitely not helping here – this task may resemble looking for a needle in a haystack, if the objective is to find a small correlated pattern.

How many biclusters? The larger gets the volume of the data, the greater is the number of potential solutions. Usually biclustering methods yield either the requested number or (by default) up to 100 of biclusters. Some methods, however, may return even millions of patterns. From the perspective of the endpoint user, performing analysis or validation of that many candidates becomes extremely challenging. As each scenario varies, we believe this should be up to the user to determine a reasonable number of biclusters that would be suitable for their purpose. One of the unexplored caveats of biclustering is that the number of expected biclusters actually influences which patterns should be indicated as biclusters, what is especially visible in partially overlapping scenarios. If the method does not account for the user indication, we recommend reporting biclusters with the highest relevance before the others (e.g. by maintaining a ranked list of the best solutions), so that the most important solutions were not missed if the user requests only a couple of biclusters, as well as filtering out highly overlapping biclusters.

How to measure the performance? The most established measures in the field, called recovery and relevance, are based on Jaccard index and were shown to be inadequate for objective assessment of the performance of biclustering methods. Horta and Campello reviewed different measures for biclustering and presented their desirable properties [9]. Biclustering measures should increasingly penalize noisy entries or elements not found in both biclusterings. Not covering all solutions, reporting elements not belonging to a specific bicluster, as well as covering the same elements multiple times should be penalized as well. Reporting repetitively a very similar bicluster is also not desirable. Finally, the measure should be symmetric and return score equal to 1 for a perfect fit. Although the authors reported two measures that have the desired properties, only Clustering Error (CE) [10] doesn't penalize heterogeneous patterns, which are very common in genomics. Thus, we believe that CE should be considered as the most objective measure of performance of biclustering methods for all synthetic scenarios.

Interpretability is the key

In the biomedical domain, interpretation is performed using expert knowledge. Gene set enrichment analysis, or pathway analysis are common techniques of validation. One of the major advantages of biclustering over many other methods (e.g. feature selection) is interpretability of the results. Biclusters are much easier to interpret as they extract very specific patterns, for detection of which the given method was designed [8]. Interpretability greatly increases understanding.

There exists a visible tendency to overinterpret the statistical significance and importance of p-values. It needs to be remembered that statistical significance doesn't imply clinical relevance, no matter which significance threshold is used. Thus, even if p-values associated with a bicluster in one method are smaller than in the other, it doesn't necessarily mean that the method is performing better. Similarly, the higher percentage of significantly enriched biclusters does not imply superiority, as this number might have been inflated by large overlapping biclusters. It may be useful to perform filtering of the returned results, or to compare the results with a random detection scenario.

Conclusions

Although some very powerful techniques have already been developed for big data, there is still a very high demand for scalable methods that can provide interpretable insights. One such technique is biclustering, which looks for local associations in data. Biclustering has previously proven its usefulness, especially in biomedical sciences.

With the recent progress in the development of highly scalable solutions, biclustering is on a good track of becoming one of the standards of big data analytics.

Abbreviations

CE: Clustering Error; CPU: central processing unit; EBIC: evolutionary search-based biclustering; GPU: graphics processing unit; RNA-seq: RNA-sequencing; scRNA-seq: single cell RNA-sequencing; TPU: tensor processing unit

Competing interests

The authors declare that they have no competing interests.

Funding

This work was supported by NIH grant LM012601.

Author contribution

Original draft preparation: P.O.; review and editing: K.B. and J.H.M.

References

1. Kasim A, Shkedy Z, Kaiser S, Hochreiter S, Talloen W. Applied biclustering methods for big and high-dimensional data using R. CRC Press; 2016.
2. Yoon S, Nguyen HCT, Jo W, Kim J, Chi SM, Park J, et al. Biclustering analysis of transcriptome big data identifies condition-specific microRNA targets. Nucleic acids research 2019;.
3. Orzechowski P, Sipper M, Huang X, Moore JH. EBIC: an evolutionary-based parallel biclustering algorithm for pattern discovery. Bioinformatics 2018 05;34(21):3719–3726.
4. Orzechowski P, Moore JH. EBIC: an open source software for high-dimensional and big data analyses. Bioinformatics 2019;p. btz027.
5. Gomez-Vela F, López A, Lagares JA, Baena DS, Barranco CD, García-Torres M, et al. Bioinformatics from a Big Data Perspective: Meeting the Challenge. In: International Conference on Bioinformatics and Biomedical Engineering Springer; 2017. p. 349–359.
6. Xie J, Ma A, Fennell A, Ma Q, Zhao J. It is time to apply biclustering: a comprehensive review of biclustering applications in biological and biomedical data. Briefings in bioinformatics 2018;.
7. Madeira SC, Oliveira AL. Biclustering algorithms for biological data analysis: a survey. IEEE/ACM Transactions on Computational Biology and Bioinformatics (TCBB) 2004;1(1):24–45.
8. Padilha VA, Campello RJ. A systematic comparative evaluation of biclustering techniques. BMC bioinformatics 2017;18(1):55.
9. Horta D, Campello RJ. Similarity measures for comparing biclusterings. IEEE/ACM Transactions on Computational Biology and Bioinformatics (TCBB) 2014;11(5):942–954.
10. Patrikainen A, Meila M. Comparing subspace clusterings. IEEE Transactions on Knowledge and Data Engineering 2006;18(7):902–916.

Notes

[1] https://www.genomicsengland.co.uk/about-genomics-england/the-100000-genomes-project/
[2] https://www.clinicalomics.com/topics/biomarkers-topic/biobanking/10-countries-in-100k-genome-club/
[3] https://allofus.nih.gov/
[4] http://tda.gatech.edu/software/bibench-v0.2/
[5] https://hadoop.apache.org/
[6] https://spark.apache.org/
[7] https://github.com/EpistasisLab/ebic

Cover letter

Click here to access/download;Personal Cover;submission-letter-gigascience-2.pdf

Perelman School of Medicine
UNIVERSITY of PENNSYLVANIA

Institute for Biomedical Informatics

Jason H. Moore, PhD
Edward Rose Professor of Informatics
Director, Institute for Biomedical Informatics
Director, Division of Informatics
Senior Associate Dean for Informatics

April 15, 2019

Dear Editors,

Following our previous correspondence, attached please find our commentary on scalable biclustering. In the paper "Scalable biclustering – the future of big data exploration?" we focus on the recent advances in biclustering. We also share our previous experience in developing scalable methods and discuss major challenges that biclustering faces from big data perspective.

Biclusering is a well-established unsupervised machine learning technique which detects subsets of rows and subsets of columns that are associated with each other. For almost 20 years, since its first application to genomic data, hundreds of different approaches emerged. Very few approaches however focused on data scalability.

With increasing volume of biomedical data, high heterogeneity, presence of noise and outliers it is crucial to foster collaborative research on developing next-generation biclustering techniques that are both accurate and scalable. One of the recent advances in the field is development of Evolutionary-search based Biclustering (EBIC), a multi-GPU parallel biclustering method that outperformed multiple approaches in terms of accuracy. The method was further improved to work on big data in 2019.

Thank you for your consideration.

Sincerely,

Jason H. Moore, Ph.D.
Director, Penn Institute for Biomedical Informatics

D202 Richards Building, 3700 Hamilton Walk • Philadelphia, PA 19104-6116 • Tel: (215) 573-4411 • Fax: (215) 573-3111
Email: jhmoore@upenn.edu

Response to the Reviewers Click here to access/download;Personal Cover;response.pdf

Response to the Reviewers

Patryk Orzechowski, Krzysztof Boryczko, and Jason H. Moore

June 7, 2019

Response to Reviewer #1

The manuscript is a nice, light read as a first time introduction to the concept of biclustering. On the other hand, the article only scratches the surface of the topic, making no attempt to give either the theory of nor detailed case study examples of biclustering. Overall the manuscript is well organized and clearly written.

We thank the Reviewer for a positive feedback. We completely agree that many aspects in the manuscript were either barely mentioned or not fully covered. The main reason for this is the limitations in the size and number of the references of the commentary.

To address the comments, we have expanded a "What is biclustering?" section by providing a formal definition of biclustering. We have also reorganized a paragraph (former paragraph in common myths section) into a separate section "What is the application of biclustering?". The section was expanded by mentioning several biomedical case studies. Additional information with more detailed coverage of use cases could be found in one of the references.

The one spot where I was not entirely sure what the authors' meant was: "Additional caution needs to be taken when interpreting the performance of the methods. For example, below a certain very small threshold p-values become numerically meaningless." in the "How to measure the performance?" subsection. I think this could use clarification.

We have rephrased the paragraph and provided additional explanation.

Response to Reviewer #2

The main objective of this commentary is to advocate for the application of biclustering in big data analytics. To achieve this goal, the authors first gave a brief introduction to biclustering in a way that easy to understand and elucidated the common misunderstandings about this technique in Q&A. Major challenges in applying Biclustering were listed afterwards. This topic is in-time for promoting this powerful technique in the big data era.

We would like to thank the Reviewer for this opinion.

However, it could be further improved if the following comments are soundly fixed.
1. The authors claimed that scalable biclustering is closing the gap in applying biclustering in big data, but there is no description of what "scalable" means. For readers without a computational background, it is not convincing enough to adopt this new concept.

Clarification was added in the "Scalable biclustering" section.

2. Figure 1 is impressive and informative. It would add more value if the authors could give a detailed figure legend regarding how to interpret these patterns.

We have added a more detailed description under the figure.

3. In the discussion of "Challenges of biclustering", a lot of them are open-ended. Readers would want more information to make decisions with respect to "size of biclusters", or "number of biclusters". So authors might consider citing examples from references or directing readers to specific papers.

We would like to thank again for the suggestions. We have clarified that algorithm developers should pay more attention in providing the users parameters that could adjust the method to specific purposes.

In our opinion no recommendation could be given on the size and number of biclusters, as this is problem-specific. For example, if the goal of the study is to find cohorts of patients, detection of large biclusters is encouraged, as the goal is to cover as many patients with a common characteristics as possible. However, if the goal is detection of gene regulatory network, large biclusters often contain lot of noise and such information extracted may not be very informative.

We would also like to emphasize that only the most relevant studies were referenced in the paper due to the limitation of the format of the paper.
