# Supplementary material for: Scalable biclustering — the future of big data exploration?
Source: Gigascience. 2019 Jun 28;8(7):giz078. doi: 10.1093/gigascience/giz078 (PMC6598466; doi:10.1093/gigascience/giz078)
Supplement: giz078_Response_to_Reviewer_Comments_Original_Submission [file giz078_response_to_reviewer_comments_original_submission.pdf]

Author's Response To Reviewer Comments

Close

Response to Reviewer #1
We thank the Reviewer for a positive feedback. We completely agree that many aspects in the manuscript were either barely mentioned or not fully covered. The main reason for this is the limitations in the size and number of the references of the commentary.

To address the Reviewer's comments we have expanded a "What is biclustering?" section by providing a formal definition of biclustering. We have also reorganized a paragraph (former paragraph in common myths section) into a separate section "What is the application of biclustering?". The section was expanded by mentioning several biomedical case studies. Additional information with more detailed coverage of use cases could be found in one of the references.

Additionally, we have rephrased and provided additional explanation for the paragraph on results interpretation.

Response to Reviewer #2

We would like to thank the Reviewer for reviewing our manuscript.

We have added clarification on scalable biclustering and created ''Scalable biclustering" section.

A more detailed description was added in the figure caption.

We have clarified that algorithm developers should pay more attention in providing the users parameters that could adjust the method to specific purposes. In our opinion no recommendation could be given on the size and number of biclusters, as this is problem-specific. For example, if the goal of the study is to find cohorts of patients, detection of large biclusters is encouraged, as the goal is to cover as many patients with a common characteristics as possible. However, if the goal is detection of gene regulatory network, large biclusters often contain lot of noise and such information extracted may not be very informative.

We would also like to emphasize that only the most relevant studies were referenced in the paper due to the limitation of the format of the paper.

Close
