# Supplementary material for: Scalable biclustering — the future of big data exploration?
Source: Gigascience. 2019 Jun 28;8(7):giz078. doi: 10.1093/gigascience/giz078 (PMC6598466; doi:10.1093/gigascience/giz078)
Supplement: giz078_Reviewer_2_Report_Original_Submission — Jing Zhao -- 5/23/2019 Reviewed [file giz078_reviewer_2_report_original_submission.pdf]

Reviewer Report

Title: Scalable biclustering - the future of big data exploration?

Version: Original Submission **Date:** 5/23/2019

Reviewer name: Jing Zhao

Reviewer Comments to Author:

The main objective of this commentary is to advocate for the application of biclustering in big data analytics. To achieve this goal, the authors first gave a brief introduction to biclustering in a way that easy to understand and elucidated the common misunderstandings about this technique in Q&A. Major challenges in applying Biclustering were listed afterwards. This topic is in-time for promoting this powerful technique in the big data era. However, it could be further improved if the following comments are soundly fixed.
1. The authors claimed that scalable biclustering is closing the gap in applying biclustering in big data, but there is no description of what "scalable" means. For readers without a computational background, it is not convincing enough to adopt this new concept.
2. Figure 1 is impressive and informative. It would add more value if the authors could give a detailed figure legend regarding how to interpret these patterns.
3. In the discussion of "Challenges of biclustering", a lot of them are open-ended. Readers would want more information to make decisions with respect to "size of biclusters", or "number of biclusters". So authors might consider citing examples from references or directing readers to specific papers.

Level of Interest

Please indicate how interesting you found the manuscript: Choose an item.

Quality of Written English

Please indicate the quality of language in the manuscript: Choose an item.

Declaration of Competing Interests

Please complete a declaration of competing interests, considering the following questions:

- Have you in the past five years received reimbursements, fees, funding, or salary from an organisation that may in any way gain or lose financially from the publication of this manuscript, either now or in the future?
- Do you hold any stocks or shares in an organisation that may in any way gain or lose financially from the publication of this manuscript, either now or in the future?
- Do you hold or are you currently applying for any patents relating to the content of the manuscript?

- Have you received reimbursements, fees, funding, or salary from an organization that holds or has applied for patents relating to the content of the manuscript?
- Do you have any other financial competing interests?
- Do you have any non-financial competing interests in relation to this paper?

If you can answer no to all of the above, write 'I declare that I have no competing interests' below. If your reply is yes to any, please give details below.

I declare that I have no competing interests.

I agree to the open peer review policy of the journal. I understand that my name will be included on my report to the authors and, if the manuscript is accepted for publication, my named report including any attachments I upload will be posted on the website along with the authors' responses. I agree for my report to be made available under an Open Access Creative Commons CC-BY license (http://creativecommons.org/licenses/by/4.0/). I understand that any comments which I do not wish to be included in my named report can be included as confidential comments to the editors, which will not be published.

I agree to the open peer review policy of the journal

To further support our reviewers, we have joined with Publons, where you can gain additional credit to further highlight your hard work (see: https://publons.com/journal/530/gigascience). On publication of this paper, your review will be automatically added to Publons, you can then choose whether or not to claim your Publons credit. I understand this statement.

Yes Choose an item.
